# The end of HIV: Still a very long way to go, but progress continues

**DOI:** 10.1371/journal.pmed.1002466

**Published:** 2017-11-30

**Authors:** Steven G. Deeks, Sharon R. Lewin, Linda-Gail Bekker

**Affiliations:** 1 Department of Medicine, University of California, San Francisco, San Francisco, California, United States of America; 2 Peter Doherty Institute of Infection and Immunity, University of Melbourne, and Royal Melbourne Hospital, Melbourne, Australia; 3 Department of Infectious Diseases, Alfred Hospital and Monash University, Melbourne, Australia; 4 Desmond Tutu HIV Centre, University of Cape Town, Cape Town, South Africa

## Abstract

In an Editorial accompanying *PLOS Medicine*’s Special Issue on Advances in Prevention, Treatment and Cure of HIV/AIDS, Guest Editors Steven Deeks, Sharon Lewin, and Linda-Gail Bekker discuss priorities in the field and the content of the issue.

*Let’s End It* is the theme of this year’s World AIDS Day, which falls on December 1. In the spirit of the event, *PLOS Medicine* is devoting this special issue to a discussion on advances in HIV prevention, treatment, and cure. Here, we describe many of the remaining barriers in ending the epidemic and highlight a number of accompanying studies that provide paths forward for overcoming some of these challenges.

An estimated 75 million people have acquired HIV infection since the first reports of infection in the 1970s. Over 35 million people have died. With the advent of combination antiretroviral therapy (ART) in 1996, the life expectancy of HIV-infected adults in both high- and low-income countries now approaches that of the general population but only in people who start therapy early in the disease process, who take therapy on a daily basis, and who have life-long access to drugs and monitoring of antiviral effects. The remarkably successful and ongoing global effort to provide treatment means that 20.9 million of the estimated 36.7 million people living with HIV globally are now receiving therapy [1]. ART not only improves health but it makes a person noninfectious; HIV-infected mothers on effective ART rarely transmit HIV to their infants, and HIV-infected adults on effective ART have been shown to not transmit the virus to their sexual partners. The significance of these achievements cannot be overstated: in the past 3 decades, global biomedical and public health programs not only discovered how HIV causes disease and developed effective strategies to prevent and treat the infection, but also built a global public health response that is unprecedented in its scale and effectiveness.

ART is the mainstay of treatment for people with HIV infection, and a crucial component of UNAIDS’s global aims to achieve, by 2020, high proportions (90%) of people, respectively, tested for HIV infection, receiving ART, and with viral suppression (the so-called 90-90-90 initiative) [2]. Despite massive international efforts to achieve this goal, many challenges remain, particularly as many of the key affected populations are highly stigmatized and marginalized. In this issue of *PLOS Medicine*, various experts address aspects of the challenges facing infants [3], children and adolescents [4], female sex workers [5–7], transgender women [8], men who have sex with men (MSM), and people who inject drugs [9].

In a Perspective on these issues, Wafaa El-Sadr and colleagues discuss the important topic of differentiated service delivery by which process interventions are combined and blended as appropriate for individual populations and settings [10]. As an example, Margaret McNairy and colleagues report on a cluster-randomized trial done in Swaziland [11] testing a combination intervention including point-of-care CD4 testing, prompt initiation of ART, and supportive components intended to improve adherence. The trial’s results show substantial benefits in the trial’s primary endpoint of linkage to and retention in care at 1 year, indicating the prospects for customized interventions to achieve the UNAIDS targets in HIV care. Batya Elul and colleagues performed a cluster-randomized trial in Mozambique and found that a similar intervention improved linkage to care and retention at 1 year [12].

Harsh political realities can also be a barrier; this is perhaps best illustrated in the intensifying crisis that is the HIV epidemic in the Russian Federation, discussed in a Perspective by Chris Beyrer and coauthors [13]. Here, as occurred in other countries earlier in the global epidemic with disastrous results, HIV infections are multiplying in key populations that lack societal attention and are denied proven healthcare interventions. In the Russian Federation and other regions where anachronistic policies and laws in relation to injection drug use and MSM fuel the spread of HIV, immediate action is needed to reverse the growth of local epidemics.

Biomedical methods to prevent HIV acquisition have undergone a resurgence in research and development in the last decade with the demonstration that ART given either as pre-exposure prophylaxis (for an uninfected individual) or as treatment (for an infected individual) can prevent transmission. Combination prevention packages are now being tailored for different populations; these approaches often take a more holistic approach and include harm reduction for people who inject drugs and social protection for young unemployed women.

Despite these advances, it is generally assumed that an effective vaccine will be needed to ultimately end the spread of HIV. In another Perspective, Lynn Morris and Nonhlanhla Mkhize discuss the prospects for broadly neutralizing antibodies (BNAbs) to contribute to HIV prevention efforts by eliciting passive immunity [14], and Ken Mayer and colleagues report a phase 1 trial of VRC01, a BNAb that is proceeding to large-scale evaluation [15]. It is hoped that such BNAb studies might identify novel therapies for prevention and provide important insights for the development of more scalable vaccines. Whether by generating active or passive immunity, supplementing the available prevention strategies with an effective vaccine remains of critical importance in circumventing the issues of adherence that affect many current approaches, with potentially transformative benefits in specific settings and populations.

For those who can access ART, residual concerns persist. For example, for reasons that remain largely undefined, HIV-infected adults on otherwise effective therapy have an excess risk of developing a number of non-AIDS complications. These complications include cardiovascular disease and kidney disease, and in a study based on the D:A:D collaboration, Mark Boyd and colleagues describe a multiplicative increase in events in people at high risk of cardiovascular and renal disease [16], which has implications for long-term management.

Due in part to problems in retaining people in care and the apparent inability of ART to fully restore health [16], there is now a major global scientific effort to find a cure for HIV disease. Although ART prevents HIV from replicating, it does not eliminate a stable reservoir of infected cells that persists indefinitely. Approaches to reducing this reservoir include starting ART very early (before the reservoir is established) or reconstructing a new HIV-free immune system through hematopoietic stem cell transplantation. Timothy Henrich and colleagues report an extraordinary case of what happened when ART was initiated on essentially the first day an infection might be diagnosed, estimated to be roughly 10 days post-infection [17]. The reservoir of replication-competent virus in this individual was several orders of magnitude lower than that observed when ART was started during chronic infection, but unfortunately the person was not cured as, even during this brief period of time, approximately 200 stably infected cells were established. A similar state of prolonged time to rebound, once ART was stopped, was achieved in an HIV-infected individual who received an allogeneic stem cell transplantation, as demonstrated by Andrew Badley and colleagues [18]. For the initial 10 months off ART, virus was undetectable but eventually rebounded. Careful sequencing of the rebound virus did not match virus sequenced in blood prior to transplantation, highlighting the great challenge in understanding the main source of viral rebound off ART. Furthermore, it is now clear that simply reducing virus, even by several logs, will not lead to durable remission without ART.

The aspirational slogan *Let’s End It* suggests that the goal of ending the epidemic is in our grasp and hinges only on our collective commitment to do so. However, the remarkable progress, activism, resources, ingenuity, and sheer fortitude that have brought us this far will be needed in at least equal measure to take us to the end. Only by harnessing the maximum available resources; innovating and implementing relentlessly; and applying the fruits of these processes without prejudice to all human populations, wherever they are needed, will we be able to start imagining an end to the HIV/AIDS epidemic.

## Abbreviations

ART: antiretroviral therapy
BNAb: broadly neutralizing antibody
MSM: men who have sex with men
